# Notes and News

**Published:** 1901-11-30

**Authors:** 


### Notes and News.

The King has intimated his pleasure that the Prince of Wales's Hospital Fund for London, established by His Majesty when Prince of Wales, shall henceforth be known as King Edward's Hospital Fund for London, and that the change of name will not come into effect until January 1st.

The Duke of Fife, K.T., has promised to preside at a festival dinner in aid of Charing Cross Hospital Special Appeal Fund at the Hôtel Cecil on March 19th, 1902.

Dr. Goodhart will deliver the Purvis Oration before the West Kent Medico-Chirurgical Society at the Royal Kent Dispensary, Greenwich, on December 6. The subject of the oration will be "General Practice and Original Research."

The Volunteer Medical Staff Corps Relief Fund amounted to £293 14s. 10d., which, with a donation from the Woolwich Companies sent direct to Aldershot, make a total of £314 15s. 9d. The whole amount collected, with the exception of the cost of postage and stationery, was handed over to the Royal Army Medical Corps Depôt Mobilisation Fund. The committee acknowledge the assistance of the editors of the *British Medical Journal*, the *Lancet*, and The Hospital, for the gratuitous insertion of advertisements and acknowledgment of donations.

The belief that plague infection in Europe is nearly entirely carried by rats should call for the extermination of these rodents both in ships and at all seaports, if nowhere else. Fighting with rats is not like fighting with anything so minute as the mosquito, and if this latter is not regarded as an impossibility, certainly rats should have but a small chance of existence. There are places where the presence of rats is regarded as a matter of course, though they may not be exactly popular. Now they are shown to be a danger to the community, and their extermination must be regarded as an absolute duty. With their disappearance it may transpire that some other scavengers may have to be employed, for even rats have their use.

The Borough Fever Hospital at Plaistow has been extended. It now can accommodate 179 patients.

A post-mortem examination of Czolgosz's body showed that the brain was perfectly healthy and rather above the average standard.

The Finsen Light treatment has been introduced into the Birmingham and Midland Hospital for Skin and Urinary Troubles.

Sir James Blyth has placed two farms near Stanstead, in Essex, at the disposal of the Royal Commission on Tuberculosis, for experimental purposes.

The Prince of Wales's Hospital Fund has received the fourth annual grant from the London Parochial Charities for the Maintenance of Convalescent Hospitals.

Mr. J. Pierpont Morgan has promised £5,000 towards the renovation and rebuilding of Guy's Hospital, when contributions by others amounting to £100,000 for this purpose have been reached. The scheme is estimated to cost £180,000.

At the meeting of the Executive Committee of the General Medical Council on Monday last an Order in Council, applying Part II. of the *Medical Act* to the island of Malta having been read, it was resolved "That the requirements for the Medical Degrees of the University of Malta being such as in the opinion of the Committee furnish a sufficient guarantee of the possession of the requisite knowledge and skill for the efficient practice of medicine, surgery, and midwifery, these degrees be recognised as entitling to registration in the Colonial List." The degree of Doctor of Medicine and Surgery in all Italian universities is also to be recognised as entitling to registration in the Foreign List of the *Medical Register*.

The Blue-book resulting from the labours of the departmental committee of the Local Government Board appointed to inquire into preservatives and colouring matters in food is now published. On the whole, the conclusions are more satisfactory than could be expected. The preservative agents principally in use are boric acid and its compounds, sulphurous acid and sulphites, the soda salt of salicylic acid, and formalin or formaldehyde. The use of boric acid is found variably prejudicial in different individuals, but in any quantity it sets up chronic dyspepsia in adults. The committee recommend that, if the use of boracic acid is permitted, the quantity should be definitely limited, and that if introduced to any article of food the fact must be stated by the seller. The use of formaldehyde and its preparations they would have absolutely forbidden. In the case of colouring matter, the verdict is mainly against tinned peas, the green of which can be secured by the use of copper sulphate. Other colouring substances in use are mostly harmless in the quantities introduced.

